# Qualitative Proteome-Wide Analysis Reveals the Diverse Functions of Lysine Crotonylation in *Dendrobium huoshanense*

**DOI:** 10.3389/fpls.2022.822374

**Published:** 2022-02-16

**Authors:** Jing Wu, Xiaoxi Meng, Weimin Jiang, Zhaojian Wang, Jing Zhang, Fei Meng, Xiaoyan Yao, Mengjuan Ye, Liang Yao, Longhai Wang, Nianjun Yu, Daiyin Peng, Shihai Xing

**Affiliations:** ^1^College of Pharmacy, Anhui University of Chinese Medicine, Hefei, China; ^2^Institute of Traditional Chinese Medicine Resources Protection and Development, Anhui Academy of Chinese Medicine, Hefei, China; ^3^Department of Horticultural Science, University of Minnesota, Saint Paul, MN, United States; ^4^Hunan Key Laboratory for Conservation and Utilization of Biological Resources in the Nanyue Mountainous Region, College of Life Sciences and Environment, Hengyang Normal University, Hengyang, China; ^5^School of Integrated Chinese and Western Medicine, Anhui University of Chinese Medicine, Hefei, China; ^6^Synergetic Innovation Center of Anhui Authentic Chinese Medicine Quality Improvement, Hefei, China; ^7^Anhui Province Key Laboratory of Research and Development of Chinese Medicine, Hefei, China

**Keywords:** *Dendrobium huoshanense*, lysine crotonylation, polysaccharide biosynthesis, heavily-crotonylated, photosynthesis

## Abstract

The lysine crotonylation of histone proteins is a newly identified posttranslational modification with diversified cellular functions. However, there are few reports on lysine crotonylation of non-histone proteins in medicinal plant cells. By using high-resolution liquid chromatography–mass spectrometry (LC-MS) coupled with highly sensitive-specific immune-affinity antibody analysis, a whole crotonylation proteome analysis of *Dendrobium huoshanense* was performed. In total, 1,591 proteins with 4,726 lysine crotonylation sites were identified; among them, 11 conserved motifs were identified. Bioinformatic analyses linked crotonylated proteins to the drought stress response and multiple metabolic pathways, including secondary metabolite biosynthesis, transport and catabolism, energy production and conversion, carbohydrate transport and metabolism, translation, and ribosomal structure and biogenesis. This study contributes toward understanding the regulatory mechanism of polysaccharide biosynthesis at the crotonylation level even under abiotic stress.

## Graphical Abstract

Outline of contents in the study.

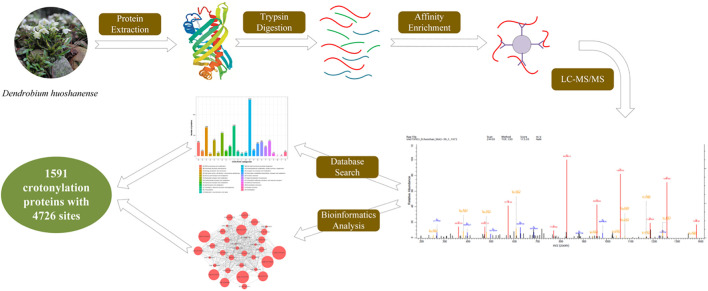

## Introduction

*Dendrobium huoshanense*, an edible and medicinal species of family Orchidaceae, was traditionally and popularly called “mihu” and has been claimed to maintain health and prolong life in the Supplement to Compendium of Materia Medica (Zhao, 2007). Famous as a tea material, soup ingredient, and Chinese herbal medicine, *D. huoshanense* can relieve immune deficiencies, improve eyesight, nourish the stomach, and produce body fluid (Bao et al., 2001). A study on plants of the genus *Dendrobium* showed that alkaloids and polysaccharides were their main bioactive compounds (Chen and Guo, 2001). Polysaccharides of *D. huoshanense* exhibit antiglycation and immunoregulatory activities (Pan et al., 2013; Zha et al., 2014), while its alkaloids are anticataract and neuroprotective (Ng et al., 2012). However, harsh growth habits and overexploitation resulted in *D. huoshanense* becoming endangered and its low yield does not meet its demand. The genome of *D. huoshanense* has recently been sequenced to promote molecular genetics and functional genomics studies (Han et al., 2020). Besides, several studies focusing on the transcriptome of *D. huoshanense* have shown differences in the main active ingredients across tissues (Yuan et al., 2018; Zhou et al., 2020). However, there had been no studies reporting effects in the proteome and protein posttranslational modifications (PTMs) of *D. huoshanense*.

Posttranslational modification, a crucial mechanism aiming at cellular regulation, is attributed to the proteolytic cleavage or addition of a specific group to one or more amino acids (Allfrey et al., 1964; Walsh et al., 2005). As an efficient strategy to modify and expand the properties of 20 natural amino acids by introducing the new groups, there is no denying that PTM plays a vital role in accurate protein folding, molecular structure, as well as physical or chemical properties (Millar et al., 2019). PTMs occur in any stage of a life cycle of a protein, entailing the increase of variance from a genome to proteome level (Doyle and Mamula, 2001). In fact, the vast majority of proteins in cells require modifications to perform their corresponding functions. The PTM procedure is precisely modulated, so that the protein shows a definite and stable or dynamic function in the cell at specific moments (Olsen and Mann, 2013). An increasing number of PTM types have attracted research attention, evolving from small chemical modifications, such as lysine acetylation (Kac) and phosphorylation, to intricate modifications, such as ubiquitylation (Huang et al., 2014). Xue et al. (2018) reported a comprehensive analysis of lysine acetylation and its various functions in rice. In rice, the lysine of histone H3 would be acetylated in response to cold stress stimuli. Kac can regulate biological processes (BPs) to protect plants growing normally. After that, succinylation was detected to be the most enriched modification in strawberry stigma, being involved in regulating AP2/clathrin-mediated vesicular transport (Fang et al., 2018). Another PTM, phosphorylation, was predicted in milk fat globule membrane in human colostrum and mature milk, with a key role in infant development (Yang et al., 2020). Meng et al. (2019) showed that N-glycoproteome proteins in duck egg white can have antibacterial effects. Altogether, many cellular BPs are known to be regulated by PTMs through their impact on signaling, gene expression, protein stability and interactions, and enzyme kinetics (Friso and van Wijk, 2015).

**FIGURE 1:**
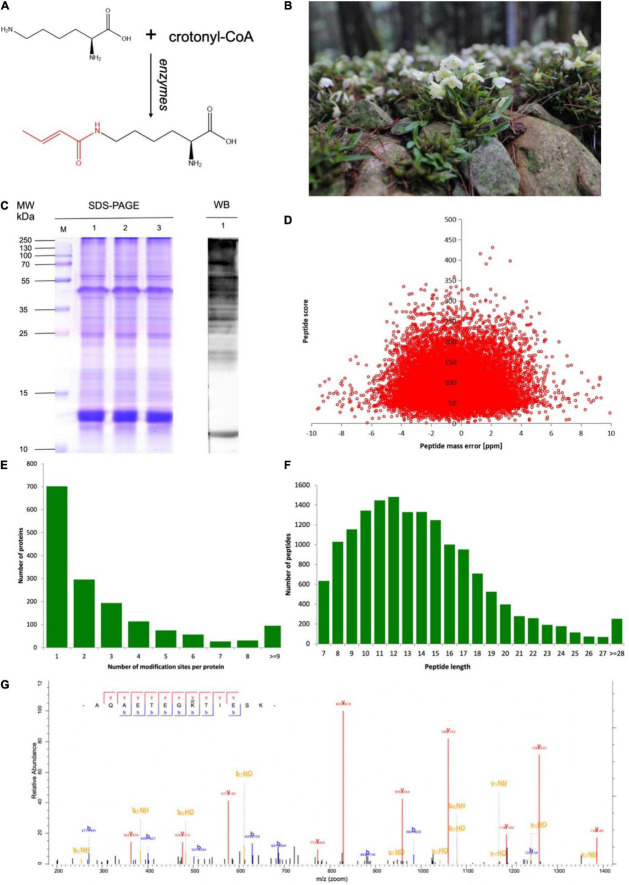
Overview of lysine crotonylation in developing *Dendrobium huoshanense*. **(A)** Structure of lysine crotonylation. **(B)** The picture of an adult *D. huoshanense*. **(C)** Western blotting screening of *D. huoshanense* lysine crotonylome. Molecular weight is labeled on the left. Samples are labeled on the top. 1, 2, and 3 on the left are images of sodium dodecyl sulfate-polyacrylamide gel electrophoresis (SDS-PAGE) stained with Coomassie brilliant blue. 1 on the right is Western blot image. Same amounts of proteins (20 μg per lane) were loaded for samples. **(D)** The mass quality precision distributions of crotonylation profiles. **(E)** Distribution of lysine crotonylation sites in one protein. **(F)** The peptide length distributions of crotonylation profiles. **(G)** The mass spectrometry (MS/MS) crotonylated protein spectrum of 60S ribosomal protein L7a.

Besides the PTMs mentioned above, there is a newly discovered type, lysine crotonylation (Kcr), which is the addition of a crotonyl group to a lysine residue (Figure 1A). In 2011, Kcr was reported to generate a “histone code” conserved from yeast to humans, mainly enriched in active promoters or potential enhancers (Tan et al., 2011). Kcr takes place at the ε-amino group in lysine and can be catalyzed by Kac enzymes, such as P300 (Sabari et al., 2015). The sizes of the four carbons and planar orientation of Kcr render it distinguishable from acetylation (Baumann, 2015). Liu et al. (2018b) analyzed histone crotonylation of rice whole-genome DNA and found a positive correlation between the degree of protein modification and gene expression. Li et al. (2016) proposed that H3 can also upregulate gene expression in the AF9 YEATS domain. Based on evidence that histone crotonylation contributes to chromatin and gene expression regulation, Kcr was revealed to widely exist in non-histones, with other biological functions besides epigenetic regulation (Xu et al., 2017). From then on, crotonylation sites were detected in myofilament and ribosomal proteins in zebrafish embryos, the human lung adenocarcinoma H1299 cells, and cervical cancer HeLa cells and had an impact on many metabolic pathways (Wei et al., 2017; Xu et al., 2017; Kwon et al., 2018). There have been abundant studies of Kcr in animals and microorganisms. Plants can quickly change their habits to adapt to the changes in their ecological environment. PTM is a more rapid and effective strategy than gene transcription and translation (He et al., 2016). However, Kcr in plants had only been analyzed in rice (Liu et al., 2018b), tobacco (Sun et al., 2017), papaya (Liu et al., 2018a), tea plants (Sun et al., 2019), *Chrysanthemum* (Lin et al., 2021), and peanut (Xu et al., 2021) and was found to contribute to a range of processes, including photosynthesis, glycolysis, and amino acid synthesis.

As a plant with high edible and medicinal value, Kcr in *D. huoshanense* has not been investigated. Therefore, we explored the comprehensive Kcr proteome of *D. huoshanense* by using high-resolution liquid chromatography–mass spectrometry (LC-MS/MS) and high-sensitive immune-affinity purification. Conservative motifs, the Gene Ontology (GO) enrichment, and the Kyoto Encyclopedia of Genes and Genomes (KEGG) were also implemented to predict the modified proteins. Our research is the first report on qualitative proteome-wide analysis of Kcr profiling in *D. huoshanense* and compares crotonylated proteins and motifs with those in tea, rice, etc., to obtain orthologous proteins and conserved motifs. Several crotonylated proteins were found to be associated with active compound biosynthesis and response to stress. This offers not only a fundamental basis for exploring the physiological role of Kcr in *D. huoshanense* and PTMs in non-histones, but also a significant resource for future studies on the molecular mechanism of plant regulation by PTMs.

## Materials and Methods

### Chemicals and Plant Materials

Plants originating from Huoshan County, Anhui Province, China were identified as *D. huoshanense* C. Z. Tang et S. J. Cheng by Professor Nianjun Yu from Anhui University of Traditional Chinese Medicine (Tang and Cheng, 1984). The whole plant of *D. huoshanense* consisted of roots, stems, leaves, and flowers (Figure 1B). Three independent 3-year-old whole plants had been collected for the samples, which were washed with ddH_2_O for 30 min to eliminate potential microbes.

### Protein Extraction

Three replicates of whole plants were fully grinded by liquid nitrogen into powders in precooled mortar, mixed, and transferred to a new centrifuge tube. Three volumes of phenolic extraction buffer [10 mM dithiothreitol (DTT), 1% protease inhibitor cocktail, 3 μM trichostatin A, together with 50 mM nicotinamide] were added to the centrifuge tube, followed by ultrasonication. Then, Tris-saturated phenol (pH 8.0) was added in drops with an equal volume into the centrifuge tube for centrifugation (4°C, 10 min, 5,000 *g*). Then, the upper phenol phase was removed to a new centrifuge tube for protein precipitation (at least five volumes of ammonium acetate-saturated methanol were added), followed by protein incubation at −20°C overnight. Centrifugation at 4°C for 10 min was carried out to discard the supernatant. Ice-cold methanol was added to polish the remaining pellet once, accompanied by ice-cold acetone three times. Last, the protein was redissolved in 8 M urea and the concentration was determined by using the bicinchoninic acid (BCA) kit by following the methods of the manufacturer.

### Protein Trypsin Digestion

For digestion, an appropriate amount of standard protein and 20% trichloroacetic acid (TCA) was dropped slowly into the protein and mixed thoroughly. The supernatant was discarded after precipitation for 2 h (4°C) and centrifugation for 5 min (4,500 *g*). Dry precipitation was diluted by adding 200 mM triethylammonium bicarbonate (TEAB) to urea, to reach a concentration less than 2 M, which was reduced with 5 mM DTT for 30 min at 56°C and alkylated with 11 mM iodoacetamide (IAA) for 15 min at room temperature in the dark. Finally, trypsin was added at a ratio of 1:50 trypsin-to-protein for digestion overnight.

### Western Blot Analysis

Phenol-extracted proteins (20 μg) from *D. huoshanense* were separated by using 15% sodium dodecyl sulfate-polyacrylamide gel electrophoresis (SDS-PAGE) and electroblotted onto a polyvinylidene fluoride (PVDF) membrane for Western blot analysis. Crotonylated proteins were detected by primary anti-crotonyllysine antibody (PTM-502, Cat. No. 1037267K306, PTM Biolabs, Hangzhou, China) in a 1:1,000 (v/v) dilution and secondary antibody goat antimouse immunoglobulin G (IgG) (31430, Thermo Fisher Scientific, Waltham, MA, United States) in a 1:10,000 dilution.

### High-Performance Liquid Chromatography Fractionation and Affinity Enrichment

Through reverse-phase high-performance liquid chromatography (HPLC) with high pH by using a Thermo Betasil C18 column (5 μm particles, 10 mm ID, and 250 mm length), the tryptic peptides were first separated with a gradient of 8–32% acetonitrile (pH 9.0) over 60 min into 60 fractions. Then, peptides were dried by vacuum centrifuging.

To enrich crotonylated peptides, trypsin digested peptides were dissolved in NETN buffer (100 mM NaCl,1 mM EDTA, 50 mM Tris–HCl, 0.5% NP-40, pH 8.0) and incubated with prewashed anti-crotonyl lysine antibodies (PTM-503, Cat. No. #TAK901B06, PTM Biolabs, Hangzhou, China) at 4°C overnight with gentle shaking. Then, beads were carefully washed four times with NETN buffer and twice with distilled H_2_O. The bound peptides were eluted from the beads with 0.1% trifluoroacetic acid. Finally, the eluted fractions were combined and vacuum dried. The resulting peptides were desalted with C18 ZipTips (Millipore, Burlington, MA, United States) according to the instructions of the manufacturer and then subjected to LC-MS/MS.

### Quantitative Proteomic Analysis by Liquid Chromatography–Mass Spectrometry

Followed by loading onto a reversed-phase analytical column (15 cm length, 75 μm id), the tryptic peptides were dissolved in solvent A, comprised of 0.1% formic acid and 2% acetonitrile. The gradient was comprised of an increase from 7 to 24% solvent B (0.1% formic acid in 98% acetonitrile) over 42 min, 24–32% in 12 min and climbing to 80% in 3 min, and then holding at 80% for the last 3 min, all at a constant flow rate of 450 nl/min on an nanoElute Ultra Performance Liquid Chromatography (UPLC) system. The intact peptides and fragments were subjected to NSI source followed by the timsTOF Pro MS coupled online to the UPLC, whose electrospray voltage was set at 1.75 kV. The first mass m/z scan range was 100–1,700 for a full scan, in which intact peptides were detected in the Orbitrap at a resolution of 70,000. Peptides were then selected for MS/MS by using the NCE setting at 28 and the fragments were detected in the Orbitrap at a resolution of 17,500. The scanning range of secondary MS was set to 100–1,700 m/z by using a parallel accumulation serial fragmentation (PASEF) mode to acquire data. A data-dependent procedure that alternated between one MS scan followed by 20 MS/MS scans with 24 s dynamic exclusion was used. Automatic gain control (AGC) was set at 5E4 and 100 m/z was set for fixed first mass.

### Mass-Spectrum Quality Control Analysis

The first-order mass error of most spectra was under 10 ppm, which was in line with the high-precision characteristics of Orbitrap MS. It showed that the qualitative and quantitative analysis of proteins would not be affected due to excessive mass deviation. The score of the matched peptides in the spectrum (indicating the credibility of peptide identification) had a negative correlation with the distribution of quality deviation (the higher the score, the smaller the quality deviation).

### Database Search

MaxQuant (version 1.6.15.0) was accessed to process raw data files acquired by the mass spectrometer and tandem mass spectra data were searched against the transcriptome database^1^ of *D. huoshanense* uploaded by us that is concatenated with the reverse decoy database and a common pollution database. With four missing cleavages, trypsin/P was used as the cleavage enzyme. For precursor ions, the mass tolerance was set to 20 and 4.5 ppm first and final stages of the search, respectively. As for fragment ions, the mass error was set to 10 ppm. Carbamidomethylation on cysteine was specified as a fixed modification, while crotonylation on lysine was specified as variable modification. False discovery rate (FDR) thresholds for proteins, peptides, and modification sites were set at 1%.

### Protein Annotation and Enrichment Analysis

The GO, provided by the UniProt-GO annotation (GOA) database,^2^ is a widespread group of evidence-based combinations between terms from the GO data and UniProtKB proteins (Dimmer et al., 2012). Converting identified protein IDs to UniProt IDs is the first step, followed by mapping to the GO ID. If not successful, the InterProScan software would be used to annotate the GO function of a protein based on a protein sequence alignment method. Molecular function (MF), cellular components (CCs), and BPs are three categories of the GO analysis.

The KEGG is a database to annotate the pathways in which proteins participate. All the identified proteins were mapped to a pathway in the KEGG database^3^ by using Blastx/Blastp. First, the KEGG Automatic Annotation Server (KAAS) (KEGG online service tool) was used to annotate the KEGG database description of a protein (Moriya et al., 2007). Then, the KEGG mapper was used to map the annotation to the KEGG pathway database.

Protein domain functions were annotated by the InterProScan (a sequence analysis application) based on a protein sequence alignment method (Zdobnov and Apweiler, 2001). InterPro^4^ database was used for integrating various information about protein families, domains, and functional sites.

We also used WoLF PSORT, a subcellular localization prediction software (Horton et al., 2007). As for functional enrichment of the GO/pathways/domains of the Kcr protein clusters, the two-tailed Fisher’s exact test was employed against the National Center for Biotechnology Information (NCBI) non-redundant protein database. The GO/pathways/domains with an adjusted corrected *p*-value < 0.05 were considered to be significantly enriched (Huang et al., 2009).

### Motif Analysis

To specify the sequence model proximal to the crotonylated residues, we examined the relative frequencies of amino acids 10 positions upstream and downstream of Kcr sites by MoMo (motif-x algorithm), modification motifs prediction software. All the protein sequences in the database were treated as background controls. The minimum number of occurrences was set to 20, *p*-value ≤ 0.0000001. The option “Emulate original motif-x” was selected and other parameters were set to default.

### Protein–Protein Interaction Analysis

The interaction network of proteins can help us to clarify the functions and mechanism of Kcr proteins. Based on the STRING database version 10.5, all the differentially expressed modified protein database accessions or sequences in this study were searched for Protein–protein interactions (PPIs). The STRING treats confidence score as a metric to define whether the interaction is real. Only interactions with a confidence score >0.7 (high confidence) were visualized by using R package “networkD3” for a network of PPIs.

### Conservative Analysis

To determine the degree of evolutionary conservation of crotonylation, BLASTP was applied for comparing crotonylated protein sequences of *D. huoshanense* against specific protein sequences from four species: *Dendranthema grandiforum* (PXD010297), *Camellia sinensis* (PXD011610), *Oryza sativa* (PXD008716), and *Nicotiana tabacum* (IPX0000889000). By applying a reciprocal best BLAST hit approach, we determined orthologous proteins among these plants.

## Results

### Proteome-Wide Identification of Lysine Crotonylation Sites in *Dendrobium huoshanense*

To detect the global distribution of Kcr in *D. huoshanense*, the trypsin-hydrolyzed protein was identified by a combination of high-specific Kcr pan antibody and high-resolution LC-MS/MS. The pan anti-crotonylated antibody helped to detect proteins modified by using SDS-PAGE and Western blot (Figure 1C). Here, 4,726 crotonylation sites derived from 1,591 proteins were quantifiable with high confidence (Supplementary Table 1). A total of 13,765 peptides were identified by trypsin digestion, among which, 4,695 peptides were crotonylated. Original data of all the identified crotonylated peptides are shown in Supplementary Table 1. The MS proteomics data have been deposited into the ProteomeXchange Consortium *via* the PRIDE partner repository with the dataset identifier PXD028293.^5^ The MS data were validated by referring to the mass error of all the identified peptides; most of them had a less than 5 ppm mass error (Figure 1D). With a diverse distribution of crotonylation sites in *D. huoshanense* proteins, 702 were crotonylated on one site, 296 were crotonylated on two sites, and 153 proteins contained 7 or more crotonylation sites (Figure 1E). Consistent with the properties of tryptic peptides, the lengths of most identified peptides varied from 7 to 28 amino acid residues (Figure 1F). In the study of 69,323 spectra generated from the mass spectrometer, 4,341 matched with protein alignments. A representative mass spectrum of a crotonylated protein [60S ribosomal protein L7a (RPL 7a)] is shown in Figure 1G.

### Functional Annotation and Subcellular Localization of Lysine Crotonylated Proteins

**FIGURE 2:**
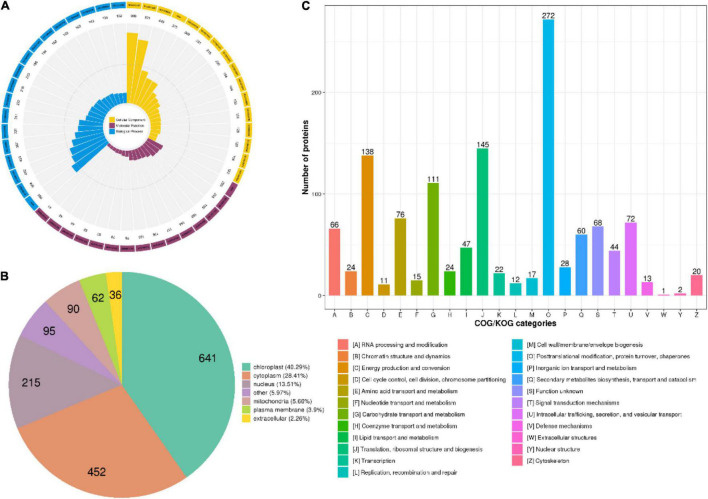
Classification of proteins in *D. huoshanense* corresponding to identified sites. **(A)** The Gene Ontology (GO) classification of the crotonylated proteins in *D. huoshanense* based on biology process, cellular components, and molecular functional. **(B)** Subcellular localization chart of proteins corresponding to modification sites. **(C)** COG/KOG functional classification of crotonylated proteins.

The crotonylated proteins identified were classified based on the GO annotation (Figure 2A, Supplementary Figure 1, and Supplementary Table 2). Metabolic processes are essential life activities in plants, whose products are important active elements for growth. Just as the importance of polysaccharides and alkaloids in *D. huoshanense*. Therefore, the top “biological process” of the GO terms was metabolic process. For example, the number of proteins in cellular metabolic processes, organic substance metabolic processes, and primary metabolic processes was up to 504, 482, and 423, respectively. In the category of “molecular function,” the majority of crotonylated proteins were involved in “binding,” which mainly consisted of “organic cyclic compound binding” (205 proteins), “heterocyclic compound binding” (199 proteins), and “ion binding” (165 proteins). Under the “cellular components” category, “cytoplasm” and “organelle” were predicted to be the most relevant GO terms in modified proteins, 909 and 831 proteins in each, respectively. The analysis of subcellular localization clarified that a majority of crotonylated proteins were mainly located in the chloroplast (40.29%), cytoplasm (28.41%), and nucleus (13.51%), suggesting that crotonylated proteins were broadly distributed in leaves and stems of *D. huoshanense* (Supplementary Table 2). Moreover, 5.66% modified proteins were mitochondria located. Among crotonylated proteins, 3.9 and 2.26% modified proteins were predicted to be localized in the plasma membrane and extracellular space, respectively (Figure 2B). We also analyzed function classifications of proteins corresponding to crotonylated sites based on the Clusters of Orthologous Groups (COGs) the COG database. A total of 272 proteins were predicted to have the function of “postmodification, protein turnover, chaperones.” “Translation, ribosomal structure, and biogenesis” possessed the second highest amount of identified proteins (145), while 138 modified proteins were predicted to be associated to “energy production and conversion” (Figure 2C and Supplementary Table 2).

### Functional Enrichment Analysis of Lysine Crotonylated Proteins

**FIGURE 3:**
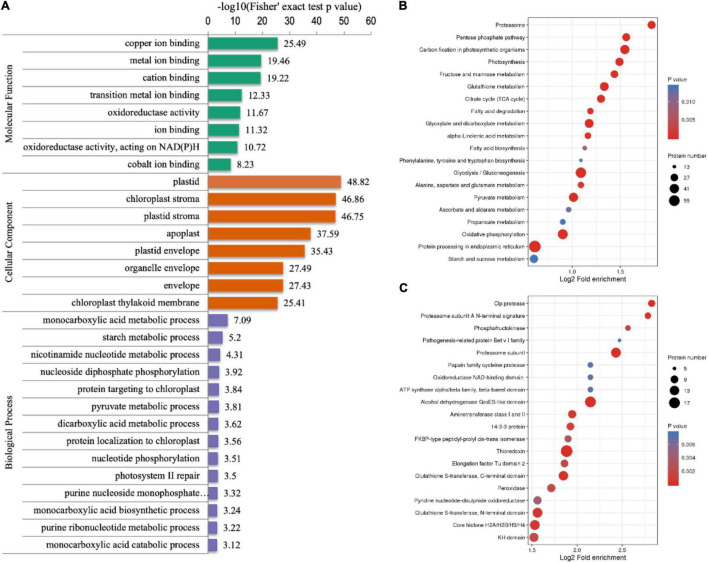
The GO classification, the Kyoto Encyclopedia of Genes and Genomes (KEGG) pathway and protein domain analyses of crotonylated proteins of *D. huoshanense*. The *p*-value obtained by the Fisher’s exact test showed the functional classification and pathway of enriched proteins. The results of the first 20 classifications most enriched were given in the bubble plot. In the bubble plot, the vertical axis is the functional classification or pathway and the horizontal axis value is the Log2 conversion value of the proportion of modified proteins in this functional type compared with the proportion of identified proteins. The circle color indicates enrichment significance and the circle size indicates the number of modified proteins in the functional class or pathway. **(A)** The GO enrichment bubble plot of proteins corresponding to modification sites in three categories: biological process, molecular function, and cellular component. **(B)** The KEGG pathway enrichment of proteins corresponding to modification sites. **(C)** Protein domain enrichment bubble plot.

To explore the roles that crotonylated proteins play, the functional enrichment analysis of identified proteins was operated based on the GO, the KEGG pathways, and protein domain annotations (Supplementary Table 3). Regarding the “biological process” category of the GO enrichment, the most well-represented terms were “monocarboxylic acid metabolic process,” “starch metabolic process,” and “nicotinamide nucleotide metabolic process.” In the “cellular components,” crotonylated proteins related to “plastid,” “chloroplast stroma,” and “plastid stroma” accounted for the largest portion. Based on the GO annotation, proteins with a “binding” function occupied a large portion, which included “copper ion binding,” “metal ion binding,” and “cation binding.” There were also many proteins predicted to be enriched in “transition metal ion binding” and “oxidoreductase activity” (Figure 3A). The KEGG pathways enrichment analysis showed “carbon fixation in photosynthetic organisms,” “proteasome,” “pentose phosphate pathway,” “photosynthesis,” and “glyoxylate and dicarboxylate metabolism” as having highest enrichment score (Figure 3B). As it is verified in protein domain enrichment analysis, “proteasome subunit,” “alcohol dehydrogenase GroES-like domain,” and “thioredoxin” were predicted to be significantly enriched among modified proteins (Figure 3C).

### Motifs and Secondary Structures of Lysine Crotonylated Peptides

**FIGURE 4:**
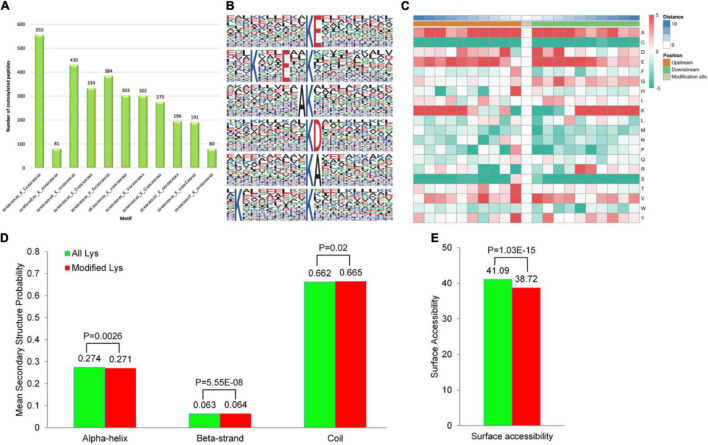
Properties of the lysine crotonylation sites in *D. huoshanense*. **(A)** The peptides length distribution in 10 consensus sequence motifs that were significantly enriched in K residues from unique Kcr-containing sites in all. **(B)** Sequence probability logos of the first six enriched crotonylation site motifs around the lysine crotonylation sites. **(C)** Heat map of the amino acid compositions around the Kcr sites showing the frequency of different types of amino acids around this residue. Red indicates enrichment and green indicates depletion. **(D)** Probabilities of lysine crotonylation in different protein secondary structures (alpha helix, beta-strand, and disordered coil). **(E)** Predicted surface accessibility of crotonylation sites.

To identify whether specific sequence motifs exist proximal to the K residues, we examined the relative frequencies of amino acids centered around each K residue (Supplementary Table 4). By using Motif X, we identified ten motifs flanking the K residues from unique Kcr-containing sites. The 10 consensus sequence motifs were KcrE, K…E…Kcr, AKcr, KcrD, KcrA, K…Kcr, KcrV, KcrG, VKcr, Kcr…E, and Ykcr, among which, the KcrE motif was present in the largest number of crotonylated peptides (555 peptides), whereas the YKcr motif was the least abundant in crotonylated peptides (80 peptides) (Figure 4A). Kcr indicates the crotonylated lysine, while E, A, D, V, G, and Y indicate glutamic acid, alanine, aspartic acid, valine, glycine, and tyrosine, respectively. These motifs are likely to represent a sequence preference for crotonylation in *D. huoshanense*. Hence, the first six motifs that were highly scored were selected for bioinformatic analysis (Figure 4B). Specifically, the GO, the KEGG, and domain function enrichment analyses on the preference of the first six significant motifs were performed. Remarkably, different motifs preferred to harboring diverse functions, as reflected on the various GO categories, pathways, and protein domains (Supplementary Figures 2–6). KcrE and KcrD were both distributed to an ATP-dependent peptidase activity (MF category), while glycogen catabolism (BP category) and apoplast and extracellular region (CC category) tended to harbor KcrD and KcrA motifs (Supplementary Figures 2–4). According to the KEGG enrichment, the first six motifs belonged to carbon fixation in photosynthetic organisms, but KcrD was enriched in protein processing in the endoplasmic reticulum (Supplementary Figure 5). Some proteins had various protein domains, illustrated by peptidase inhibitor I9 showing AKcr and K…Kcr motifs and core histone H2A/H2B/H3/H4 showing K…E…Kcr and KcrD motifs (Supplementary Figure 6).

A hierarchical cluster analysis of the frequencies of various residues related to Kcr was also performed on these motifs. As shown in the heat map (Figure 4C), the enrichment of A and E residues was mainly observed in the −5 to +5 positions and residue D was markedly enriched in the −3 to +3 position. While the K residue was abundant in the −10 to −5 and +5 to +10 positions, the C residue was not observed. We found that the occurrence of KcrE, K…E…Kcr, AKcr, and KcrD was comparatively higher than that of other conserved motifs. In accordance with the discovery, it can be inferred that crotonylation in *D. huoshanense* was preferred on K residues adjacent to glutamic acid, alanine, and aspartic acid.

Proteins are composed of linear sequences of amino acids, but only acquire their corresponding activity and biological function when folded into a specific spatial conformation. Thus, understanding the distribution of Kcr in the secondary structure of proteins is helpful to clarify the roles of crotonylated proteins in *D huoshanense*. The structural analysis of crotonylated proteins is shown in Figure 4D. Kcr sites preferentially occurred in disordered coils (66.5%), while only approximately 27.07% of sites occurred in α-helices and 6.42% in β-strands (Figure 4D). They also tended to occur more frequently on the protein surface, with a high relative surface accessibility of 94% (Figure 4E). Therefore, Kcr likely does not affect the surface properties of modified proteins. These results imply that crotonylation could selectively modify K residues in proteins and change their structure and activity indirectly, which may affect their functions in *D. huoshanense*.

### Protein Interaction Network of the Crotonylated Proteins in *Dendrobium huoshanense*

**FIGURE 5:**
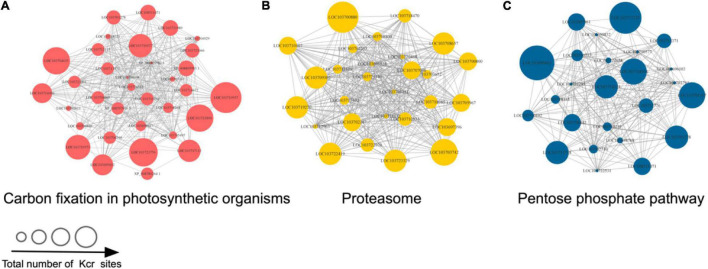
The three KEGG pathways with the highest score in the protein–protein interaction (PPI) networks of Kcr proteins in *D. huoshanense*. The network of Kcr protein interactions was visualized by using Cytoscape (listed with protein ID names). **(A)** Carbon fixation in photosynthetic organisms. **(B)** Proteasome. **(C)** Pentose phosphate pathway. The size of the dots represents the number of Kcr sites in each network.

We generated protein interaction networks of all the identified proteins by using the PPI database to clarify their function. Based on this network, crotonylated proteins associated with various interacting pathways were characterized and a large subinteraction network was further constructed and visualized by Cytoscape. According to the results of the above KEGG pathway enriched analysis, we constructed an interaction network composed of crotonylated proteins originating in three highly scored enrichment pathways, including carbon fixation in photosynthetic organisms, the proteome, and pentose phosphate pathway (Supplementary Table 5; Figures 5A–C). Proteins were detected to interact with various neighbors in these networks. All the proteins in Figure 5 are annotated in Supplementary Table 5.

Given the lack of functional information of interactions above, further research should be carried out to verify potential interactions. More bioinformatic analyses may benefit to exploring the roles of Kcr in *D. huoshanense*. Furthermore, they will be useful in selecting key proteins and their possible mechanisms.

## Discussion

### Comparison of Lysine Crotonylation Among *Dendrobium huoshanense* and Other Plants

Although great progress has been made in the study of crotonylation in microorganisms (Li et al., 2016; Yang et al., 2018), animals (Ruiz-Andres et al., 2016), and humans (Kelly et al., 2018; Wan et al., 2019), Kcr research in plants had been in its infancy. However, there was evidence showing that crotonylation possesses a certain association to plant growth and development. With the innovation of high-resolution MS and highly specific antibodies, research on plant crotonylation has been greatly promoted, such as in peanut (Xu et al., 2021), papaya (Liu et al., 2018a), *Chrysanthemum* (Lin et al., 2021), tea (Sun et al., 2019), rice (Liu et al., 2018b), and tobacco (Sun et al., 2017; Table 1). However, there were no reports for crotonylation in *D. huoshanense*. This study identified 4,726 Kcr sites in 1,591 modified proteins that were involved in diverse growth activities. Crotonylated proteins and sites in different plants lead to different consequences. Peanut (*Arachis hypogaea*) showed 6,051 sites in 2,580 proteins (the largest in amount), while the minimum number of proteins (637) containing 2,044 sites was identified in tobacco. On the contrary, what all the plants had in common was their first three categories of subcellular localization. Crotonylated proteins in peanut, papaya, *Chrysanthemum*, tea, rice, tobacco, as well as in *D. huoshanense*, according to ratio order of location, from high to low, were all located to chloroplast, cytosol, followed by nuclear. It was worth noting that crotonylated proteins in the seven species had an extensive abundance in chloroplasts, which is characteristic in plants. Thus, we have considerable reasons to conclude that crotonylation is conserved in the same locations of different plant species.

**TABLE 1:** The number of crotonylated proteins and sites identified of *Dendrobium huoshanense* compared with previous studies of plants.

Species	Tissues	Kcr proteins	Kcr sites	References
*Arachis hypogaea*	Leaves	2,580	6,051	Xu et al., 2021
*Carica papaya*	Fruits	2,120	5,995	Liu et al., 2018a
*Dendranthema grandiforum*	Leaves	1,199	2,017	Lin et al., 2021
*Camellia sinensis*	Leaves	971	2,288	Sun et al., 2019
*Oryza sativa*	Leaves	690	1,265	Liu et al., 2018b
*Nicotiana tabacum*	Leaves	637	2,044	Sun et al., 2017
*Dendrobium huoshanense*	Whole plants	1,591	4,726	

**FIGURE 6:**
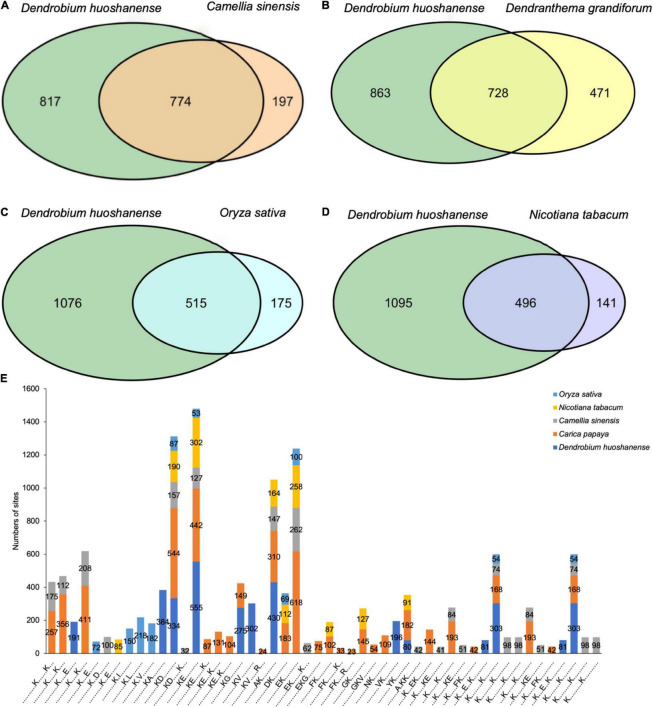
Venn diagrams of the orthologous crotonylated proteins between *D. huoshanense* and tea **(A)**, *Chrysanthemum* **(B)**, rice **(C)**, and tobacco **(D)**. **(E)** Stacked histogram of crotonylation motifs of *D. huoshanense* compared with other species.

Crotonylated protein sequences of *D. huoshanens*e were compared with specific protein sequences from tea, *Chrysanthemum*, rice, and tobacco. With a reciprocal best BLAST hit approach, we determined the orthologous proteins modified among these species. Consequently, *D. huoshanens*e had 774, 728, 515, and 496 orthologous crotonylated proteins with tea, *Chrysanthemum*, rice, and tobacco, respectively (Figures 6A–D and Supplementary Table 6). We also analyzed conserved motifs identified in *D. huoshanense* with those in other plants, finding a large number of conserved motifs (Figure 6E and Supplementary Table 6). It is worth noting that KcrD and KcrE were generally conserved in these five plants. It can be inferred that crotonylation was preferred on K residues adjacent to aspartic acid and glutamic acid in plants.

### Heavily-Crotonylated Proteins in *Dendrobium huoshanense*

Among the 1,591 proteins identified in *D. huoshanense*, heat shock protein 70 (Hsp70) displayed the most abundant crotonylated sites (25 sites). The second most heavily crotonylated protein was chaperone protein ClpB3 (21 sites), followed by elongation factor 2 (EF2) and ribulose-1,5-bisphosphate carboxylase (rbcl), both with 20 sites. Their MS/MS counts (representing the abundance of crotonylation) were 30, 25, 23, and 47, respectively.

Plants are usually exposed to various abiotic stresses, such as extreme temperatures, drought, heavy metals, and high salinity. These can bring about the imbalance of cellular homeostasis associated with a series of pathological processes (Zhu, 2002). With a disturb to plant growth and gene potential, these stresses were treated as the most challengeable problem in agriculture, which can further entail a loss of grain yield at more than 50% (Boyer, 1982; Parry and Hawkesford, 2012). It was surprising that crotonylated proteins had been proved to reduce the accumulation of reactive oxygen species (ROS) under low temperature stress and improve cold tolerance in *Chrysanthemum* (Huang et al., 2021). Hsp70, a highly conserved protein, had been reported to play a vital role in maintaining cellular homeostasis and protecting living organism against stress, such as through viral suppression (Vega et al., 2006). It is a key molecular and biochemical indicator that can be used to evaluate physiological responses to stresses (Sánchez et al., 2001). Working as a molecular chaperone and stress tolerance response protein, it assists the refolding of unfolded proteins after partial denaturation to keep protein homeostasis (Lindquist, 1986). In addition, it had also been reported that GRPs may positively enhance germination and seedling growth by regulating stomata movement when under heat and drought stresses in *Arabidopsis* (Kim et al., 2008; Koussevitzky et al., 2008). In this study, GRP-2 was shown to be crotonylated in two sites. Gamma-aminobutyric acid (GABA), with seven modified sites, had been reported to play a regulatory role in the diurnal rhythm of drought-tolerant *N. tabacum* (Pelvan et al., 2021).

When exposed to drought stress, *D. huoshanense* seedlings were often compensated by increased levels of enzymes related to adversity resistance and stress response processes. Monodehydroascorbate reductase (MDHAR), catalase (CAT), glutathione peroxidase (GPX), glutathione S-transferase (GST), superoxide dismutase (SOD), glycine-rich RNA-binding protein (GRP), ascorbate peroxidase (APX), and DHAR are ROS-scavenging enzymes that can deal with drought stress (e.g., protein damage and degradation, including ROS damage) (Koh et al., 2015). MDHAR, CAT, GPX, GST, SOD, GRP, APX, and DHAR were identified to have more than 2 Kcr sites in *D. huoshanense*.

Based on conserved protein analysis (Figures 6A–D), APX, CAT, GPX, GRP2, and GST were conserved in tea, *Chrysanthemum*, rice, and tobacco, implying that all the five enzymes were extensively crotonylated in plants. Crotonylation had been shown to positively regulate APX activity under abiotic stress, further reduce the oxidative damage caused by it Lin et al. (2021). Therefore, the crotonylation of proteins may modulate ROS stress. The decreased activities of enzymes caused by drought stress (e.g., protein damage and degradation, including ROS damage) could be compensated by increased levels of crotonylated enzymes in *D*. *huoshanense.* This deduction contributed to further analyzing the regulatory role of crotonylation in plants under environmental stress.

### Crotonylated Proteins Are Involved in the Calvin Cycle, Photosynthesis, and Alkaloid and Polysaccharide Biosynthesis Metabolic Pathways

**FIGURE 7:**
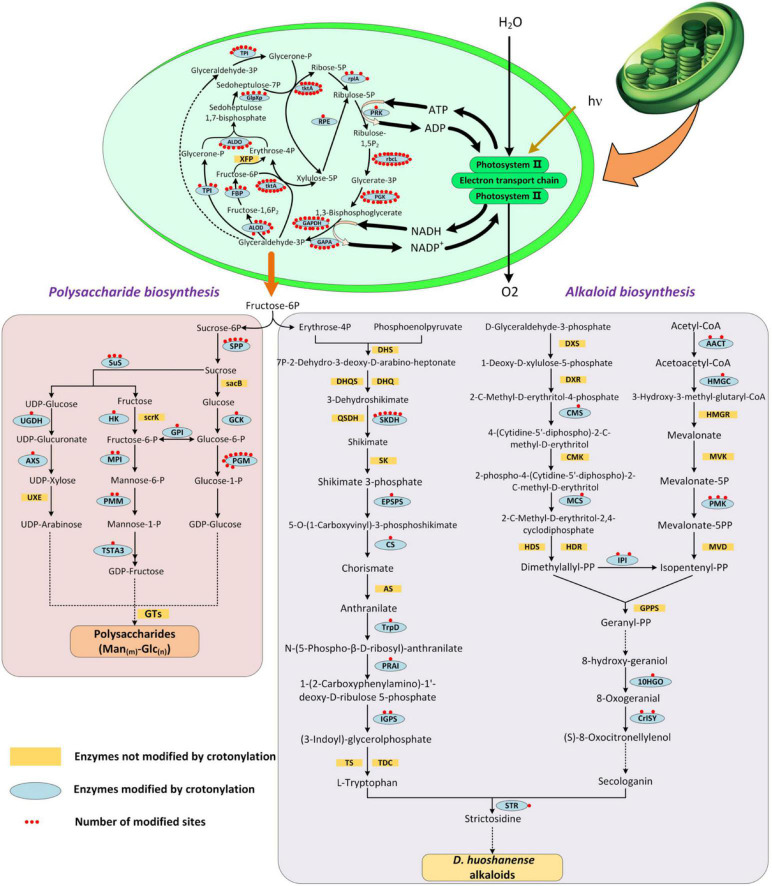
Crotonylated enzymes were involved in Calvin cycle, polysaccharide biosynthesis, and alkaloid biosynthesis pathways of *D. huoshanense*. Yellow box referred to enzymes not modified by crotonylation. Blue oval referred to enzymes modified by crotonylation. Red dots indicated crotonylated sites in each enzyme.

During the growth period, plants synthesize a series of physiological metabolites, which accumulate to form the material basis to allow normal physiological processes to occur (Dyson et al., 2015). In *D. huoshanense*, photosynthesis metabolites and alkaloids are dominant bioactive metabolites supporting its development and medical efficacy (Li et al., 2021; Shang et al., 2021). A large proportion of Kcr proteins were predicted to be found in leaf chloroplasts. Furthermore, carbon fixation in photosynthetic organisms and photosynthesis were two of the most enriched pathways. Polysaccharides are transported from leaves to whole plants, of which bulk accumulated in carbon metabolism of chloroplast (Rennie and Turgeon, 2009; Knoblauch and Peters, 2010). Photosynthesis aims at converting solar energy into energy that can be absorbed by plants in the terms of ATP, sugars, and alkaloids (Gest, 2002). Thus, to shed light on the roles that crotonylation plays in main compound synthesis during photosynthesis, we focused on proteins related to the Calvin cycle, photosynthesis, and alkaloid and polysaccharide biosynthesis metabolic pathways (Figure 7). Surprisingly, an enormous number of proteins associated with these processes were modified with more than one site, including chloroplast-encoded large subunits (rbcl, 20 sites) and fructose-1,6-bisphosphatase (FBP) (4 sites). Ribulose-1,5-bisphosphate carboxylase/oxygenase (Rubisco) fixes CO_2_ from the atmosphere into the biosphere as the rate-limiting enzyme in the Calvin cycle forming carbohydrates (Field et al., 1998; Spreitzer and Salvucci, 2002). Rbcl is a subunit of Rubisco, encoded by the chloroplast genome (Lundqvist and Schneider, 1989). Notably, rbcl owned the most abundant counts of MS/MS among all the proteins crotonylated in this manuscript. It has been described that the more rbcl is acetylated, the less active Rubisco is (Gao et al., 2016). This negative regulation to Rubisco indicated that CO_2_ fixation efficiency can be improved through a lysine modification of rbcl (crotonylation included). FBP was detected to own four sites, being another crucial enzyme of the Calvin cycle and contributing to generating ribulose 1,5-bisphosphate (RuBP) (Serrato et al., 2009). FBP catalyzed fructose-1,6-bisphosphate (F1,6BP) into fructose-6-phosphate (F6P), which is used for synthesizing soluble sugars and starch in chloroplasts and the cytosol (Zimmermann et al., 1976). The deficiency of FBP entailed the impairment of plenty of physiological processes, such as photosynthesis and CO_2_ fixation, as revealed in *Arabidopsis thaliana* (Rojas-González et al., 2015). There were also many other enzymes tightly linked to alkaloid and polysaccharide biosynthesis, such as phosphoglycerate kinase (PGK), phosphoglucomutase (PGM), phosphomannose isomerase (PMM), and shikimate dehydrogenase (SKDH) (1, 18, 2, and 6 sites, respectively). PMM, with two sites, was involved in mannose biosynthesis, which will be further explored by our next study concerning key enzymes in *D. huoshanense* polysaccharide biosynthesis. Protein crotonylation in *D. huoshanense* plays an important role in modulating the accumulation of main active ingredients. This role may be exerted through the regulation of enzymes mediating carbon fixation and photosynthetic efficiency. *D. huoshanense* is a precious, but endangered traditional Chinese herb and it is unfortunate that the mechanisms responsible for its synthesis of active compounds are poorly understood. Here, we identified several crotonylated enzymes involved in the biosynthesis of two dominant substances, polysaccharides and alkaloids, which were also involved in photosynthesis. These two compounds were promising for medicinal development due to their wide pharmacological activities. The candidate protein information was provided not only for protein structure analysis and functional verification, but also as the basis for optimizing the breeding quality of *D. huoshanense* and the optimization of germplasm resources.

## Data Availability Statement

The original contributions presented in the study are publicly available. This data can be found here: the mass spectrometry proteomics dataset has been deposited into the ProteomeXchange Consortium *via* the PRIDE partner repository with the dataset identifier PXD028293 (http://proteomecentral.proteomexchange.org/cgi/GetDataset?ID=PXD028293).

## Author Contributions

SX, JW, and DP conceived, designed, and implemented the study. JW and ZW contributed to the statistics analysis. JW, SX, DP, NY, FM, XY, and JZ contributed to the reagents, materials, and analysis tools. SX, JW, XM, and WJ drafted the manuscript. All authors edited the manuscript and approved the final version of the manuscript.

## Conflict of Interest

The authors declare that the research was conducted in the absence of any commercial or financial relationships that could be construed as a potential conflict of interest.

## Publisher’s Note

All claims expressed in this article are solely those of the authors and do not necessarily represent those of their affiliated organizations, or those of the publisher, the editors and the reviewers. Any product that may be evaluated in this article, or claim that may be made by its manufacturer, is not guaranteed or endorsed by the publisher.

## Footnotes

1 https://pan.baidu.com/s/1bdA4oE_LjkhC41eePsv0pg, access code: 2290.

2
http://www.ebi.ac.uk/GOA/

3
https://www.genome.jp/kegg/genes.html

4
http://www.ebi.ac.uk/interpro/

5
http://proteomecentral.proteomexchange.org/cgi/GetDataset?ID=PXD028293
