# Supplementary material for: Qualitative Proteome-Wide Analysis Reveals the Diverse Functions of Lysine Crotonylation in Dendrobium huoshanense
Source: Front Plant Sci. 2022 Feb 16;13:822374. doi: 10.3389/fpls.2022.822374 (PMC8888884; doi:10.3389/fpls.2022.822374)
Supplement: Supplementary file 1 [file Data_Sheet_1.docx]

Supplementary Figures


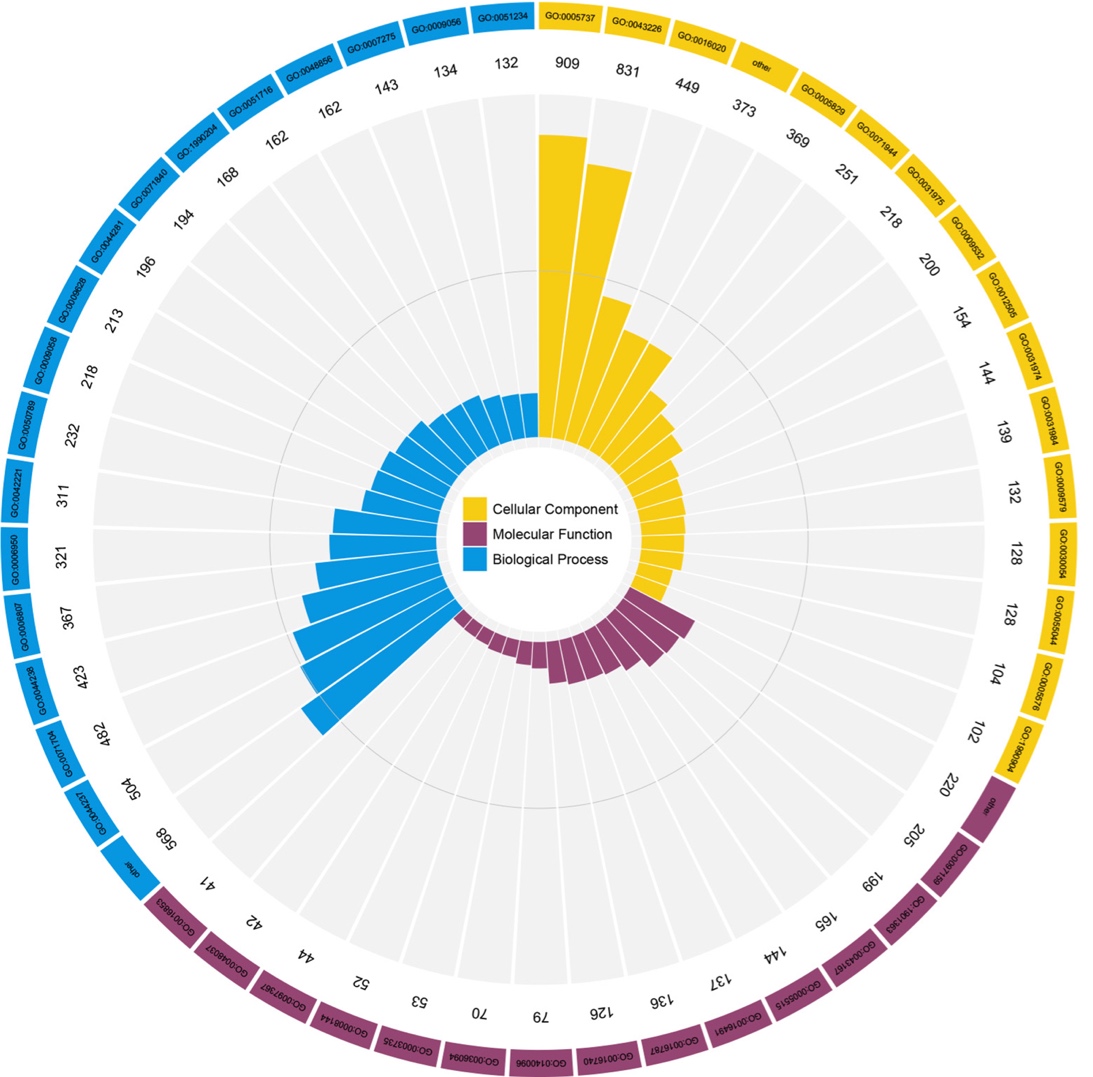


**Supplementary Figure 1** GO classification of the crotonylated proteins based on biology process, cellular components and molecular functional

According to the p value of Fisher's exact test obtained by enrichment analysis, the related functions in different groups are clustered together by hierarchical clustering method and drawn as heat map. The horizontal direction of heat map represents the enrichment test results of different groups, and the vertical direction is the description of differentially expressed enrichment related functions (GO, KEGG pathway, protein domain).

**
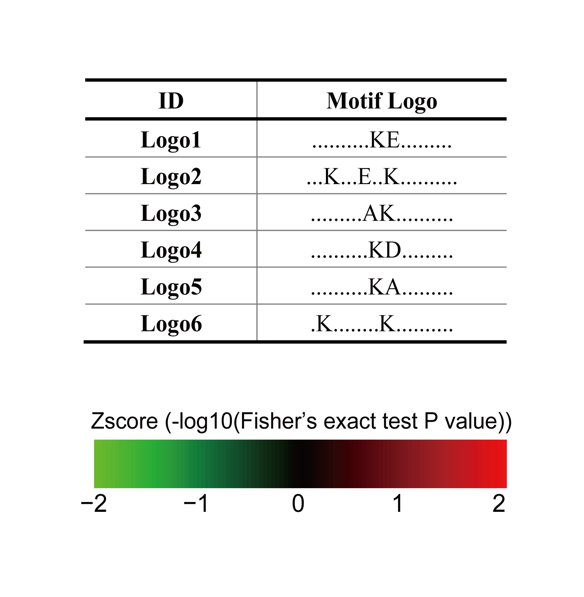
**


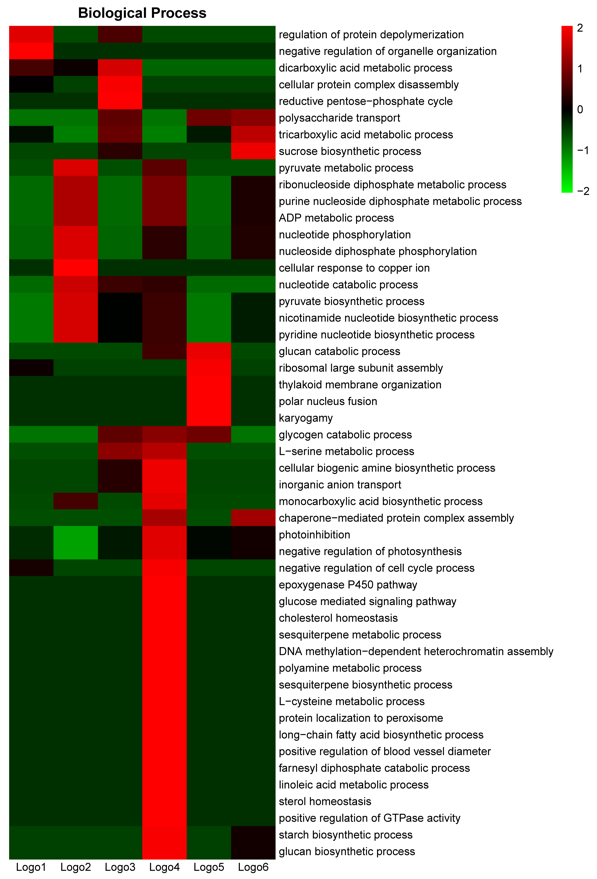


**Supplementary Figure 2** Biological Process enrichment analysis of the first six significant motifs


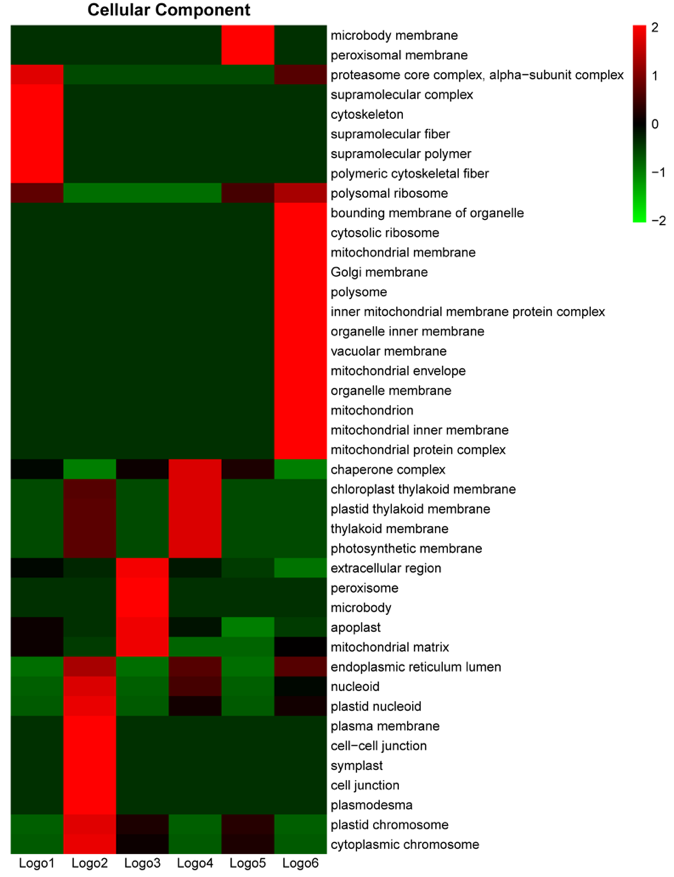


**Supplementary Figure 3** Cellular Component enrichment analysis of the first six significant motifs


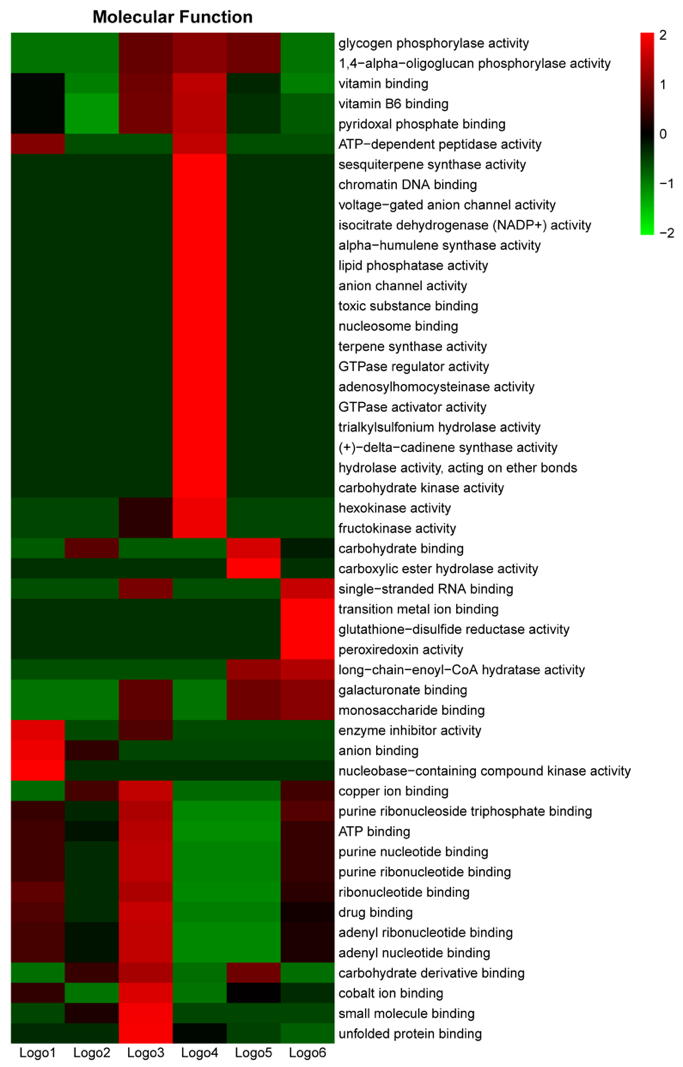


**Supplementary Figure 4** Molecular Function enrichment analysis of the first six significant motifs


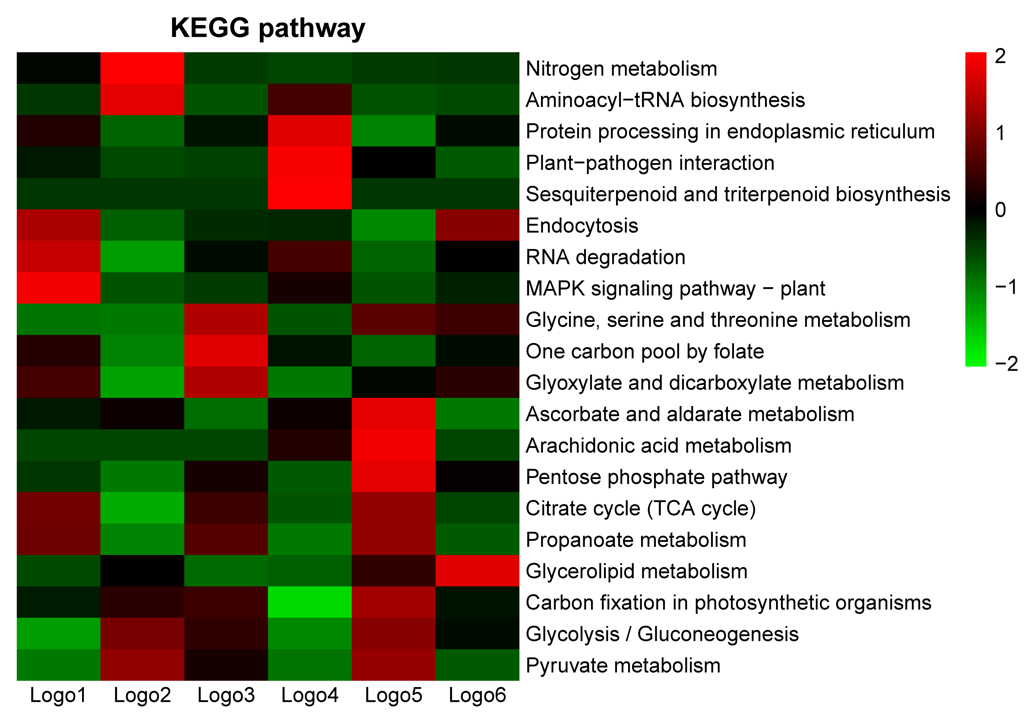


**Supplementary Figure 5** KEGG pathway enrichment analysis of the first six significant motifs


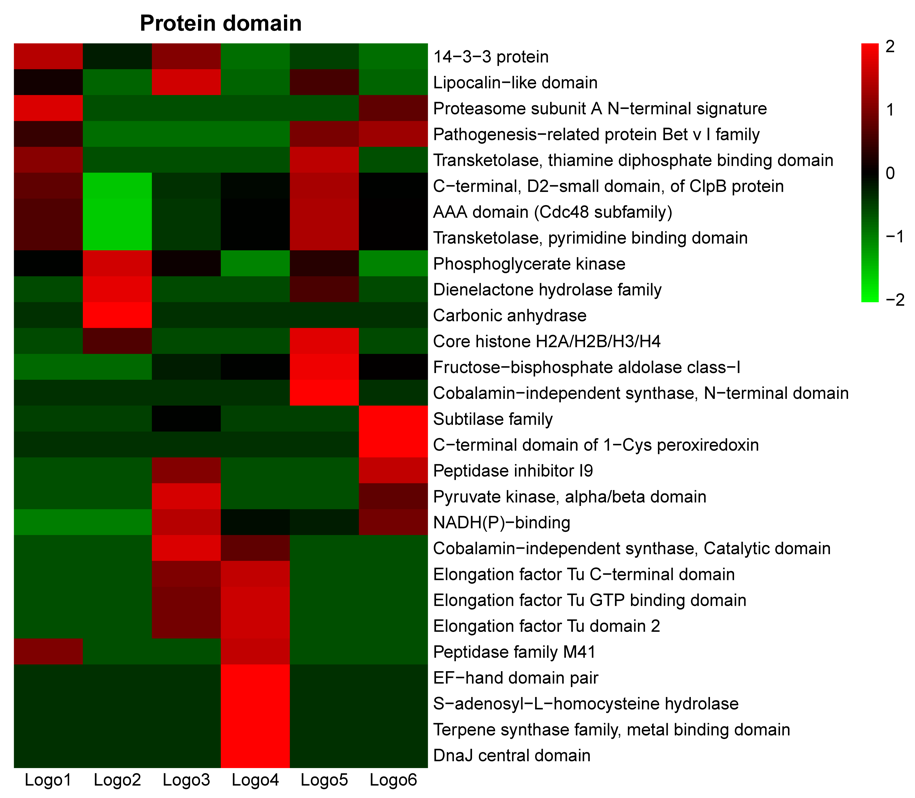


**Supplementary Figure 6** Protein Domain enrichment analysis of the first six significant motifs
